# Seasonal and Altitudinal Effects on Chemical Composition and Rumen Degradability of Blackberry Leaves in Northwestern Italian Alps

**DOI:** 10.3390/ani15010111

**Published:** 2025-01-06

**Authors:** Sonia Tassone, Salvatore Barbera, Sara Glorio Patrucco, Hatsumi Kaihara, Khalil Abid

**Affiliations:** Department of Agricultural, Forest and Food Sciences, University of Turin, Largo P. Braccini 2, 10095 Grugliasco, TO, Italy; sonia.tassone@unito.it (S.T.); salvatore.barbera@unito.it (S.B.); sara.gloriopatrucco@unito.it (S.G.P.); hatsumi.kaihara@unito.it (H.K.)

**Keywords:** blackberry leaves, chemical composition, in vitro degradability, goats, seasonal and altitudinal variations

## Abstract

The blackberry poses a threat as an invasive plant in various regions worldwide, where it aggressively competes with native species and risks delicate ecosystems. Livestock grazing has emerged as a potential strategy to mitigate its spread. This study investigated the effects of seasonal variations and altitude on the chemical composition and in vitro degradability of blackberry leaves. The leaves accessible to goats were collected in the Northwestern Italian Alps across all seasons at three altitudes (low: 450 m, medium: 700 m, high: 1000 m). The findings indicated that blackberry leaves can serve as a cost-effective, high-protein, and high-fiber feed for ruminants in these regions. However, the goats exhibited a limited capacity for fiber degradation. Furthermore, the nutritional value of these leaves was significantly influenced by the season, altitude, and their interaction. Spring leaves had the highest protein content (241.9 g/kg dry matter) and the best digestibility, with lower lignin levels (69 g/kg dry matter). At higher altitudes, lignin content decreased significantly during winter compared to the other seasons, resulting in a significant increase in fiber degradability. These insights offer crucial guidance for optimizing the utilization of the blackberry plant in goat feeding systems and underscores the necessity of considering both seasonal and altitudinal factors in grazing management practices.

## Simple Summary

The blackberry is an invasive plant that spreads rapidly, harming native species and fragile ecosystems. Controlling its growth is a challenge, but using grazing livestock like goats could be an effective solution. This study explored how the quality and digestibility of blackberry leaves change with the seasons and at different altitudes in the Northwestern Italian Alps. Leaves were collected from areas accessible to goats during all four seasons and at three heights above sea level: low, medium, and high. The results showed that blackberry leaves can be a nutritious, affordable feed option for goats, offering high protein and fiber content. However, goats struggle to break down the fiber effectively. Spring leaves were the most nutritious, with higher protein levels and better digestibility, while winter leaves from higher altitudes were easier to digest due to lower lignin levels. These findings highlight the potential of blackberry leaves in goat diets and emphasize the importance of planning grazing based on seasonal and altitude-related changes. This approach could benefit farmers by reducing feed costs and helping manage invasive plants while protecting the environment.

## 1. Introduction

The blackberry (*Rubus fruticosus*, Rosaceae) is a prickly and scrambling shrub plant known for its highly invasive nature in certain regions, where it rapidly forms thickets with dense shade canopies, aggressively outcompeting native species and posing a threat to delicate ecosystems [1]. The encroachment of shrubs presents a complex and multifaceted challenge across various environmental, social, and economic dimensions by rendering previously utilized land unsuitable [2,3,4], heightening the risk of wildfires and disrupting ecological communities, while degrading habitat quality for native flora and fauna [5,6]. In Northwestern Italy, this plant occupies a significant portion of the landscape, accounting for approximately 11% of the mesophilic vegetative cover and 3% of the overall vegetation cover [7]. Despite its invasive nature, shrub plants in mountainous regions offer economic and environmental benefits, such as the enhancement of carbon dioxide absorption and reduction of soil erosion on vegetated slopes [8,9,10].

Livestock grazing, particularly with goats, has been proposed as a potential solution to curb the spread of blackberry [11,12,13]. Goats, known for their agility and unique browsing habits, demonstrate a preference for consuming blackberry leaves, aided by their mobile lips and specialized tongues [12]. This grazing behavior not only contributes to controlling blackberry proliferation but also provides an opportunity to transform the resulting biomass into a valuable feed resource during the summer season when conventional forage availability is often limited [13].

The leaves of the blackberry exhibit considerable variability and are often utilized in veterinary medicine for their pharmacological properties, including cytotoxic, antibacterial, antioxidant, antidiabetic, and antidiarrheal effects [14,15,16]. These leaves also contain a rich array of secondary metabolites, particularly phenolic and flavonoid compounds, which peak in concentration during the summer months [17,18]. A recent innovative strategy has highlighted the potential of incorporating different feed resources rich in bioactive compounds into ruminant diets to enhance the health and vitality of animals and the quality and safety of their products [19,20]. These feed resources include grape seed, grapefruits, plum puree, Gentiana lutea root, marjoram, rosemary, sage, broccoli, pomegranate rind, pomegranate peel, orange peel, Chrysanthemum morifolium flower, hop, thyme, neem leaves, and hazelnut peel. However, despite extensive exploration of the metabolites in blackberry leaves, there remains a gap in understanding their nutritional value.

The aim of this study was to elucidate the seasonal dynamics of blackberry leaf nutrient contents across different altitudes, to guide goat farmers towards sustainable practices, and to optimize the utilization of these leaves as a sustainable alternative feed source for goats.

## 2. Materials and Methods

### 2.1. Plant Material

The leaves of blackberry were collected from the Northwestern Italian Alps at three different altitude zones (lower average: 450, middle average: 700, and upper average: 1000 m) in spring, summer, autumn and winter. The selection of these altitudes was based on their accessibility to goats and the presence of blackberry vegetation throughout the grazing seasons. The lower altitude (450 m) corresponds to the valley floor. The upper altitude (1000 m) represents the highest elevation accessible to goats during grazing seasons; altitudes above this level were excluded due to snow cover, which particularly restricts access during the winter season. The intermediate altitude (700 m) represents a transitional zone, with conditions falling between those of the valley floor and the higher elevations. The temperature and humidity data for each altitude and season are presented in Table 1.

During each season and within each of the three altitudinal zones, approximately 500 g of fresh leaves were collected at various phenological stages specific to the season: leaf development in spring, flowering in summer, fruit ripening in autumn and the dormant stage in winter. Collection was conducted randomly in marginal meadows within 3–5 designed areas per altitude zone and season, with each area covering one square meter. In each area, all leaves reachable by goats were harvested manually from shoots using scissors and immediately put in bags without additional handling.

### 2.2. Chemical Analysis and Calculations

Samples were dried at 60 °C for 24 h and ground (1 mm screen) using a cutting mill (MLI 204; Bühler AG, Uzwil, Switzerland). They were analyzed according to AOAC [21] for: dry matter (DM, #930.15), ash (#942.05), and crude protein (CP, #984.13). Fiber fractions, specifically neutral detergent fiber corrected for ash (NDFom, Method 12), acid detergent fiber corrected for ash (ADFom, Method 12), and acid detergent lignin (ADL, Method 8) were determined using the Ankom200 Fiber Analyzer (Ankom Technology, Macedon, NY, USA). The NDF of the samples was analyzed without sodium sulfite and α-amylase [22].

### 2.3. In Vitro Ruminal Degradability

The in vitro degradability was determined in three separate incubations (runs) conducted over three consecutive weeks, using goat rumen fluid as inoculum and Ankom Daisy^II^ (Ankom Technology Corporation Fairport, NY, USA) as the incubator.

Each week, rumen fluid was collected from three slaughtered female Saanen dairy goats at the end of their productive lifespans. These goats were in good health and had not been slaughtered in an emergency state. Each goat was 8 years old and weighed 50 kg, and they were daily fed with 2 kg of mixed hay from the second harvest and 1 kg of a flaked maize-based concentrate. The collection took place immediately post-slaughter in controlled conditions in the slaughterhouse. The rumen fluid was transported immediately to the laboratory within 20 min after slaughtering and maintained at 39 °C, following the protocol of Fortina et al. [23]. In the laboratory, the rumen fluid was collected, and immediately filtered with a cheesecloth fabric mesh of 250 µm porosity.

During each run, dried and ground samples were weighed (0.50 ± 0.01 g) in triplicate into Ankom F57 acetone filter bags (ANKOM Technology, Macedon, NY, USA) and heat sealed. The samples were divided into each season and put in a different fermentation jar of the incubator. Additionally, two empty Ankom filter F57 acetone filter bags, as blanks, were added to each fermentation jar and were used to correct degradability, resulting in a total of 20 Ankom F57 acetone filter bags (three altitudinal zones × six replicates + two blanks) for the fermentation jar of the Ankom Daisy^II^ incubator. Each fermentation jar was filled with 400 mL of goat-filtered rumen fluid and 1600 mL of buffer solution (ratio 1:4, vol:vol), and purged with CO_2_ for 2 min at 39 °C according to the procedure of Goering and Van Soest [24]. The fermentation jars were closed and then placed into the pre-heated Ankom DaisyII incubator at 39 °C and kept rotating for 48 h. At the end of the incubation, the Ankom F57 filter bags were removed, rinsed thoroughly with cold tap water until the water became clear, and were dried in a 60 °C forced-air oven for 24 h. The bags were weighed to determine the quantity of residue on DM and then the residue was analyzed for NDF content with the Ankom200 Fiber Analyzer (Ankom Technology Corporation, Fairport USA). After correction with blanks, the in vitro apparent and true DM degradability (ADMD_vt_, TDMD_vt_, respectively) and NDF degradability (NDFD_vt_) were calculated as follows:

ADMD_vt_ = 100 × (DM_0h_ − DM_residue_)/DM_0h_ (1)

TDMD_vt_ = 100 × (DM_0h_ − NDF_residue_)/DM_0h_ (2)

NDFD_vt_ = 100 × (NDF_0h_ − NDF_residue_)/NDF_0h_ (3)

where DM_0h_ = sample weight on dry matter ante incubation DM_residue_ = residue weight on dry matter post incubation NDF_0h_ = sample weight on neutral detergent fiber ante incubation NDF_residue_ = residue weight on neutral detergent fiber post incubation.

### 2.4. Statistical Analysis

The data were statistically analyzed using a two-way factorial treatment design in a completely randomized design SAS software (version 9.1; SAS Institute Inc., Cary, NC, USA). The general linear model used was as follows:

Y_ijk_ = µ + S_i_ + Aj + (S × A)_ij_ + ε_ijk_ (4)

where Y_ijk_ was the experimental data, µ was the overall mean, S was the effect of season, A was the effect of altitude, S × A was the effect of the interaction between season and altitude, and ε_ijk_ was the residual error. When a significant effect was detected, the differences between means were evaluated using Tukey’s test, to compare all the combinations between the two factors, altitude and season. The significant difference was declared at *p* ≤ 0.05.

## 3. Results

### 3.1. Chemical Composition

The chemical composition of blackberry leaves in spring, summer, autumn and winter at different altitudes is listed in Table 2.

The chemical composition varied greatly depending on the season, except for the ADL compounds. The highest CP content was found in leaves during spring, with values averaging 242 g/kg DM. In the same period, blackberry leaves had the lowest DM and NDFom content, averaging 220 g/kg FM and 343 g/kg DM respectively. The ADFom had the lowest values in winter (average 155 g/kg DM). The ash content was similar in the winter, spring and summer seasons and increased sharply in the autumn season, with an average increase of 29% compared to the other seasons. Considering altitude, the CP, ADFom, ADL and ash content of blackberry leaves varied greatly. The highest CP and ash content were found at higher altitudes (averaging 200 and 75 g/kg DM, respectively), while the ADFom and ADL had the lowest values (averaging 190 and 60 g/kg DM, respectively). Moreover, there was also a significant interaction between season and altitude for ADFom and ADL content. Blackberry leaves had similar ADFom content at different altitudes in spring, summer, and autumn. In the winter season, the ADFom values were lower than in other seasons and decreased significantly, especially at low and high altitudes. The ADL ranged from 48 g/kg DM in the winter season to 69 g/kg DM in the summer season at lower altitudes, compared with the ADL at intermediate altitudes, ranging from 63 g/kg DM in the summer season to 107 g/kg in the winter season, and at the lowest altitudes, ranging from 66 g/kg DM in the spring season to 98 g/kg DM in the summer season.

### 3.2. In Vitro Degradability

The in vitro degradability of blackberry leaves, assessed through ADMD_vt_, TDMD_vt_, and NDFD_vt_, is detailed in Table 3. TDMD_vt_ values ranged from 683 g/kg in summer at 700 m to 746 g/kg in spring at 450 m, showing significant seasonal and altitudinal variations, with notable interactions between season and altitude. ADMD_vt_ was highest in winter at 1000 m (506 g/kg) and lowest in autumn at 700 m (426 g/kg), similarly exhibiting significant seasonal and altitudinal variations and interactions. NDFD_vt_ demonstrated the most pronounced seasonal variation, with significant interactions between season and altitude, reaching its highest value in winter at 1000 m (291 g/kg) and its lowest in spring at 1000 m (150 g/kg).

## 4. Discussion

The scarcity of conventional feed for livestock, coupled with the inflation of their prices, has compelled nutritionists to seek alternative feed sources [25]. In mountainous regions, leaves from trees and shrubs play a crucial role as feed for ruminants. Proper management of the utilization of these plant leaves enables the maximization of their nutritional benefits, thus reducing the gap between feed demand and availability [26,27,28]. While the nutritional value of various leaves from forest plants and shrubs has been extensively studied [29,30], little information is available regarding the nutritional value of blackberry leaves [13]. This study is the first to investigate how seasonal changes, altitude, and their interactions influence the chemical composition and degradability of blackberry leaves.

### 4.1. CP Content

Blackberry leaves were characterized by a high CP content (127–257 g/kg DM) comparable to that of leguminous shrubs in the mountainous regions of Spain [29] and in the mountainous regions of Ethiopia [30]. The CP content in the blackberry leaves was principally related to seasonal variability (*p* < 0.0001), with the best CP content in spring (216–257 g/kg DM) at all altitudes, due to the initial growth of the leaves at this time of year, which is accompanied by high mitotic activity and a high demand for nutrients, especially nitrogen [31]. This CP content was higher than the daily protein requirements of ruminants, which vary between 70 and 200 g/kg DM depending on the species, sex, and physiological state [32], serving as a good protein supplement to low-protein roughage in the diet of domestic ruminants.

However, these compounds progressively decreased during the growing stages of the blackberry plant from spring to winter (in summer: 147–217 g/kg DM; in autumn:153–181 g/kg DM; in winter: 127–152 g/kg DM) due to tissue aging of the leaves, translocation of nutrients to perennial tissues before abscission [27,29], and a reduction in the photosynthetic activities and uptake of essential nutrients from the soil induced by environmental stress [30]. The dynamic seasonal variation was similar to that observed in leguminous shrub leaves in the mountainous regions of Spain [29], grasses and forbs in the Alpine mountainous regions of Greece [33], and leaves of fodder multipurpose tree species in the Himalayan mountainous regions of India [27], suggesting a common response to environmental factors. In the winter season, additional protein sources must be provided for grazing goats with a high physiological protein requirement.

On the other hand, altitude had a significant influence on the CP content (*p* < 0.05), demonstrating the lowest CP content (average 165 g/kg DM) at mid-altitude compared to those at low (average 194 g/kg DM) and high altitudes (average 200 g/kg DM). Similarly, Koidou et al. [33] stated that altitudinal zone variations significantly affected the CP content of grasses and forbs in the mountainous regions of Greece. In the winter season, the CP content increased coupled with plant height (127, 146 and 152 g/kg in the lower, middle, and upper zones, respectively), which can be explained by the physiological strategy of plants growing at higher altitudes to improve their resistance to freezing temperatures by increasing their protein content [34] and accumulating antifreeze proteins [35] or delaying the ripening process of plants [36], enabling them to better withstand low temperatures. In fact, temperatures in the upper zone can drop to −10 °C, compared to −2 °C and −3 °C in the lower and middle zones, respectively. This temperature gradient likely influences the plant’s ability to adapt and optimize its metabolic processes to prevent damage from freezing and support growth in these harsh conditions. Consequently, these adaptive strategies contribute to the observed increase in CP content at higher altitudes.

### 4.2. NDFom Content

The leaves of the blackberry can serve as an alternative fiber feed as NDFom content was an average 365 g/kg DM, which is comparable to that of leguminous shrubs in the mountainous regions of Spain [29]. Only the seasonal variations led to significant changes in their cell wall components (*p* < 0.05), contrasting with the seasonal dynamic variations of the CP content. Indeed, low NDFom content was observed in the spring season (average 343 g/kg DM) and increased during the growing season, due to the aging of the leaf tissue [29]. A comparable dynamic was observed in the leaves of legume shrubs in the mountainous regions of Spain [29] and in the herbaceous vegetation in the mountainous regions of Greece [33], while fiber compounds in the leaves of multipurpose tree species in the Himalayan mountainous regions of India were reduced in summer [27]. This difference can be explained by the different latitudes, climatic conditions and/or the type of plant.

### 4.3. ADFom Content

The ADFom compounds in blackberry leaves were variable (116–242 g/kg DM), with seasonal fluctuations exerting a pronounced variability (*p* < 0.0001). The leaves of winter had the lowest ADFom content, and the leaves of summer had the highest ADFom content (average 218–242 g/kg DM). This result is consistent with Ravhuhali et al. [28] who observed higher ADFom concentrations for tree and shrub leaves in South African rangelands during summer. In previous studies on blackberry leaves collected during the summer season in Australia, Mcgregor [13] found ADFom content from 152–176 g/kg DM, which is comparable to the ADFom content observed in winter blackberry leaves in our study conducted in Italy, taking into account that the seasons are reversed in the two hemispheres. These findings highlight the complex interplay between season and geographical location in the composition of blackberry leaves.

### 4.4. ADL Content

The ADL content in the blackberry leaves (average 76 g/kg DM) was comparable to that found by Ammar et al. [29] in the leaves of another shrub in the mountainous regions of Spain. Interestingly, unlike the other compounds, the ADL content was not affected by seasonal variations. This stands in contrast to the observed fluctuations in ADL content among leguminous shrub leaves in the mountainous regions of Spain across different seasons, as reported by Ammar et al. [29]. This difference can be attributed to the different plant species and their physiological responses. Only altitude exerts a significant effect (*p* < 0.01) on the ADL content of blackberry leaves, with lower content observed in the higher altitude zone (average 60 g/kg DM) compared to those at low and medium altitude zones (average 84 g/kg DM). This suggests a potential adaptation of ADL content to varying environmental conditions, particularly altitude gradients. Similarly, the effect detected in Kobresia littledalei in the mountainous regions of China [35], attributed to the slower metabolic activities of plant cells at lower temperatures, led to an increase in the pool of metabolites, while concurrently affecting the structural cell wall components, especially the lignification process [37,38].

### 4.5. Ash Content

The leaves of the blackberry had a moderate ash content (53–88 g/kg DM), a range comparable to the leaves of leguminous shrub in the mountainous regions of Spain (Ammar et al., 2004), which demonstrated a dependency on altitude (*p* < 0.05). Similarly, Koidou et al. [33] demonstrated that altitudinal zone variation significantly affected the ash content of grasses and forbs in Alpine mountainous regions of Greece. Leaves in both lower and upper altitude zones had a comparable ash content, with values of 73.8 and 74.4 g/kg DM, respectively, while the leaves in the middle zone had a lower ash content of 63 g/kg DM. This difference could be explained by the variance in the mineral content in the soil. Seasonal variation also had a significant impact on leaf ash content (*p* < 0.05), with the ash content of blackberry leaves higher in the autumn (82 g/kg DM) than in the other seasons (winter, spring and summer 64, 68 and 68 g/kg DM respectively). Similar seasonal trends in ash content were observed in grasses and forbs in the Alpine mountainous regions of Greece [33], leaf fodder from multipurpose tree species native to the Himalayan mountains in India [27] and the tree and shrub leaves in the rangelands in South Africa [28]. However, this trend was not evident in the leaves of the leguminous shrubs in the mountainous regions of Spain [29]. This difference could be attributed to differences in plant characteristics, suggesting that not all species respond uniformly to seasonal variations.

### 4.6. Water Content

The leaves of the blackberry had a high water content (598–811 g/kg fresh matter, FM), yet the moisture content of these leaves changed depending on the season. Similar findings were reported by Navale et al. [27] in the leaf fodder from the multipurpose tree species of the Indian Himalayan Mountains. The highest moisture content was recorded in spring (780 g/kg FM), indicating optimal hydration levels coinciding with the period of new growth and blooming flora. Consequently, as temperatures rise during the summer months, reaching 28, 32, and 30 °C at low, middle, and high altitudes, respectively, a gradual decline in moisture content was observed to 697 g/kg FM, reflecting the increased evaporation rates typical of warmer climates.

This downward trend persisted into autumn, with moisture levels further diminishing to 687 g/kg FM as the transition to cooler weather ensued. Notably, the most significant reduction in moisture content was observed during the winter season, plummeting to 596 g/kg FM. This sharp decline likely reflects the onset of freezing temperatures, which curtails water uptake by the plant, resulting in cellular dehydration and inducing the acclimation mechanism [39,40].

### 4.7. In Vitro Degradability

Although the analysis of chemical composition is important for understanding the nutritional potential of plant species, it is not sufficient. Understanding the ability of ruminants to degrade plants is an essential step in better determining their nutritional value. The in vitro method, using rumen fluid from slaughtered animals offers a welfare friendly approach that is highly correlated with the in vivo degradability method and very sensitive to the antinutritional compounds, typically found in maquis and shrub plants [26,41,42].

The DM degradability of blackberry leaves (average ADMD_vt_ and TDMD_vt_ of 472 and 709 g/kg of DM, respectively) was lower than the ADMD_vt_ and TDMD_vt_ of leaves from other shrub species collected in the mountainous regions of Spain [29] and higher than the TDMDvt of leaves from other shrub species collected in the mountainous regions of Ethiopia [30]. This difference could be attributed to significant variations in topography, altitude, rainfall distribution, soil fertility and management conditions, as well as differences in plant species [30]. McGregor [13] utilized the pepsin–cellulase method to evaluate the DM degradability in blackberry leaves collected from the mountainous regions of Australia during the summer season, reporting 709 g/kg of DM for young leaves and 701 g/kg of DM for old leaves. Despite methodological variances and geographical disparities, our results indicated the TDMD_vt_ values closely corresponded to those reported by McGregor [13], comparable to the traditional feed used in goat nutrition. Blackberry leaves had a comparable ADMD to alfalfa (Medicago sativa) hay (ADMD of 63.4%) [43]. These findings demonstrated that blackberry leaves could serve as a reliable forage resource for ruminants in various geographical locations worldwide. The degradability of DM in blackberry leaves depended on altitude zone, season, and the interaction between them. DM degradability of blackberry leaves was higher in spring (ADMD_vt_ 486 g/kg DM; TDMD_vt_ 732 g/kg DM) and in the upper zone during autumn and winter. This could potentially be offset by lower ADFom, ADL content, and higher CP content during this period, compared to the other zones in the same period. Similar results were proved by Guo et al. [35] where the in vitro rumen degradability of Kobresia littledalei was higher in the upper zone in the Alpine meadows of the Tibetan plateau (China). During this period, the higher degradability of blackberry leaves growing at higher altitudes may provide more energy to local herbivores than leaves growing at lower altitudes. Furthermore, despite potential reductions in snowfall due to climate change, snow can still occur at these altitudes during winter, affecting grazing accessibility for animals. Nonetheless, in the absence of snow cover, the winter biomass at the higher altitudes of mountains can play a pivotal role in sustaining local herbivore populations during periods of feed scarcity. Farmers can capitalize on the blackberry as a valuable nutritional resource by allowing goats to graze on the available vegetation. Implementing this approach can significantly enhance the resilience of mountain goat production systems to climate change challenges, thereby ensuring their long-term sustainability. Only in the middle altitude zone the DM degradability was significantly influenced by the seasons, increasing in spring and significantly decreasing in the other seasons. These findings are consistent with those noted by Ammar et al. [29] regarding the in vitro DM degradability of legume shrub leaves in Spain at 900 m altitude. The improved degradability in spring can be attributed to several factors. Firstly, the low fiber content of the leaves correlates negatively with the DM degradability [44]. Additionally, the reduction of the lignin compound, which is an indigestible fraction, inhibits the access of microbial enzymes to the structural polysaccharides of the cell wall [45]. Moreover, the low content of polyphenolic compounds, especially condensed tannins in the spring season [17,18], which have a negative effect on the proliferation of microbiota and the activity of rumen enzymes, forming undegradable complexes with cell-wall polysaccharides and protein in the rumen [46]. Consequently, it is advisable to utilize blackberry leaves in this zone during the spring season to provide more energy and protein.

The fiber degradability of blackberry leaves was very low and showed a high variability (150–291 g/kg DM), In comparison, the in vivo digestibility of alfalfa (Medicago sativa) hay by goats ranges from 514 to 581 g/kg DM, and for fescue (Festuca arundinacea) it ranges from 430 to 604 g/kg DM, depending on the stage of maturity [43,47]. It is significantly dependent on season (*p* < 0.05) and on the interaction between altitude and season (*p* < 0.001). At lower and medium altitudes, the leaf fibers of this plant showed higher degradability in the spring season, while their degradability progressively decreased in the other seasons, particularly in autumn. This decrease could be explained by the aging of the leaves, leading to a higher lignin content. Additionally, the increase in polyphenolic compounds in the leaves during summer, as noted by Koczka et al. [18], may further contribute to reducing fiber degradability during this period. Conversely, at higher altitudes, the degradability of fiber significantly increased in winter compared to other altitudes during the same season. This result is explained by the significant decrease in ADFom and ADL in leaves in this zone during winter.

## 5. Conclusions

This study demonstrated that blackberry leaves can serve as a potential source of protein and fiber for goats. Seasonal and altitudinal variations influence their chemical composition and in vitro degradability. In spring, the leaves exhibited higher CP content and greater degradability, presenting optimal nutritional value for grazing goats. However, during the winter months, particularly at higher altitudes (1000 m), the leaves showed favorable fiber degradability compared to other seasons and altitudes. Nonetheless, supplementation with CP is necessary during this season, as the biomass produced is insufficient to meet the requirements of highly productive animals.

## Figures and Tables

**Table 1:** Minimum and maximum temperature and humidity at different altitudes and seasons in the Northwestern Italian Alps.

Season	Altitude (m)	Temperature (°C)	Humidity (%)
Spring	450	3 to 19	65 to 75
	700	5 to 20	60 to 75
	1000	−2 to 20	60 to 85
Summer	450	13 to 28	55 to 66
	700	15 to 32	50 to 65
	1000	10 to 30	55 to 80
Autumn	450	3 to 23	70 to 75
	700	8 to 22	65 to 80
	1000	0 to 18	65 to 85
Winter	450	−2 to 8	70 to 75
	700	−3 to 8	70 to 85
	1000	−10 to 8	70 to 90

**Table 2:** The chemical composition (g/kg DM basis) of blackberry leaves during seasons at different altitudes.

Season	Altitude (m)	DM *	Ash	CP	NDFom	ADFom	ADL
Spring	450	233.4 ^cd^	66.1 ^abc^	256.7 ^a^	340.8 ^bc^	193.1 ^bc^	65.7 ^bc^
	700	237.8 ^cd^	70.8 ^bc^	215.8 ^ab^	360.1 ^abc^	206.7 ^abc^	74.9 ^abc^
	1000	189.2 ^d^	66.6 ^abc^	253.1 ^a^	328.6 ^c^	206.2 ^abc^	67.3 ^bc^
Summer	450	311.9 ^abc^	71.3 ^abc^	216.7 ^ab^	360.8 ^abc^	242.3 ^a^	98.4 ^ab^
	700	339.6 ^bc^	55.6 ^bc^	147.0 ^c^	370.4 ^abc^	218.1 ^abc^	63.0 ^bc^
	1000	257.7 ^cd^	77.6 ^ab^	214.0 ^ab^	399.4 ^a^	223.4 ^abc^	68.6 ^bc^
Autumn	450	303.6 ^abcd^	85.6 ^a^	178.0 ^bc^	366.5 ^abc^	225.9 ^abc^	86.2 ^abc^
	700	345.5 ^abc^	71.9 ^abc^	153.2 ^c^	384.1 ^ab^	229.7 ^ab^	94.4 ^ab^
	1000	290.3 ^bcd^	87.7 ^a^	181.4 ^bc^	384.4 ^ab^	212.4 ^abc^	54.3 ^c^
Winter	450	413.2 ^a^	72.4 ^abc^	126.9 ^c^	342.1 ^bc^	158.0 ^d^	83.4 ^abc^
	700	402.4 ^ab^	53.5 ^c^	145.5 ^c^	384.4 ^ab^	190.4 ^c^	107.1 ^a^
	1000	395.8 ^ab^	65.9 ^abc^	152.0 ^c^	353.3 ^abc^	116.4 ^e^	48.2 ^c^
S.E.M.		73.16	13.35	37.20	30.36	22.01	22.92
P season		<0.001	0.014	<0.001	0.026	<0.001	NS
P altitude		NS	0.033	0.026	NS	0.026	0.005
P seas × altit		NS	NS	NS	NS	0.012	NS

DM, dry matter; CP, crude protein; NDFom, neutral detergent fiber corrected for ash; ADFom, acid detergent fiber corrected for ash; ADL, acid detergent lignin; S.E.M, standard error of the mean; P seas × altit, probability season × altitude; means with different letters (a–e) along the same column differ at *p* ≤ 0.05 (Tukey’s test); NS, non-significant *p* > 0.05; * g/kg fresh matter basis.

**Table 3:** In vitro true dry matter degradability, apparent dry matter degradability and neutral detergent fiber degradability of blackberry leaves during seasons at different altitudes.

Season	Altitude (m)	TDMD_vt_	ADMD_vt_	NDFD_vt_
Spring	450	746 ^a^	490 ^ab^	254 ^ab^
	700	732 ^ab^	503 ^a^	250 ^abc^
	1000	721 ^abcd^	467 ^abc^	150 ^d^
Summer	450	718 ^abcde^	487 ^ab^	219 ^abcd^
	700	683 ^de^	435 ^cd^	162 ^d^
	1000	697 ^bcde^	487 ^ab^	215 ^abcd^
Autumn	450	722 ^abcd^	446 ^bcd^	156 ^d^
	700	678 ^e^	426 ^d^	163 ^d^
	1000	709 ^abcde^	495 ^a^	151 ^d^
Winter	450	704 ^abcde^	479 ^abc^	175 ^cd^
	700	688 ^cde^	447 ^bcd^	187 ^bcd^
	1000	724 ^abc^	506 ^a^	291 ^a^
S.E.M.		23.2	22.3	55.1
P season		0.048	0.045	<0.001
P altitude		0.023	0.002	NS
P seas × altit		0.034	0.014	0.001

TDMD_vt_, true dry matter degradability (g/Kg dry matter); ADMD_vt_, apparent dry matter degradability (g/Kg dry matter); NDFD_vt_, neutral detergent fiber degradability (g/Kg neutral detergent fiber); S.E.M, standard error of the mean; P seas × altit, probability season × altitude; means with different letters (a–e) along the same column differ at *p* ≤ 0.05 (Tukey’s test); NS, non-significant *p* > 0.05.

## Data Availability

Data will be made available from the authors upon reasonable request.
